# Germination-Induced Biofortification: Improving Nutritional Efficacy, Physicochemical Properties, and In Vitro Digestibility of Black Rice Flour

**DOI:** 10.3390/foods14162912

**Published:** 2025-08-21

**Authors:** Lingfeng Zhu, Qiutao Xie, Dandan Qin, Yi He, Hongyan Yuan, Yingchao Mao, Zhaoping Pan, Gaoyang Li, Xinxin Xia

**Affiliations:** Dongting Laboratory, Hunan Institute of Agricultural Product Processing and Quality Safety, Hunan Academy of Agricultural Sciences, Changsha 410125, China; zlfjgs@hunaas.cn (L.Z.); jiningxqt@126.com (Q.X.); qdandan94@163.com (D.Q.); heyi0615@126.com (Y.H.); yhyjgs@hunaas.cn (H.Y.); mycjgs@hunaas.cn (Y.M.); pzpjl@163.com (Z.P.); lgy7102@163.com (G.L.)

**Keywords:** germination treatment, black rice, nutritional component, α-amylase activity, morphological changes, digestibility

## Abstract

Germination is an effective strategy for enhancing functional and processing characteristics of whole grains. This research aimed to explore the changes of nutritional components, physicochemical properties, in vitro digestibility, and microstructural characteristics of black rice flour (BRF) during 0–48 h germination. The results showed that germination significantly induced α-amylase activation of BRF, from 1.02 U/g to 4.46 U/g, leading to a 3.2-fold increase in reducing sugar content through starch hydrolysis. The content of apparent amylose was down-regulated during germination. The contents of free amino acids and minerals were markedly augmented in BRF. Specially, the GABA content was remarkedly enhanced, from 40.73 mg/kg to 258.35 mg/kg. Compared with BRF, the ratio of rapidly digestible starch (RDS) and resistant starch (RS) of germinated black rice flour (GBRF) increased by 12.04% and 0.43%, respectively, while the ratio of slowly digestible starch (SDS) decreased by 12.47% at 48 h. Scanning electron microscopy (SEM) analysis observed a more porous and loose surface structure in GBRF. X-ray diffraction (XRD) analysis illustrated that the relative crystallinity of GBRF was reduced with the prolonging of germination time. The dissociation of starch granules in GBRF ultimately led to a decrease in characteristic viscosity parameters, including peak, trough, final, and setback viscosity. In conclusion, germination improved the nutritional value and digestive characteristics of BRF, and altered its structure and physicochemical properties, which provides a reference for the development of whole grain-based products.

## 1. Introduction

Black rice (*Oryza sativa* L.) is categorized as a pigmented whole grain with rich anthocyanins in the bran layer, giving it a black or dark brown color [1]. Black rice is mainly cultivated in regions such as China, India, Thailand, Vietnam, and Indonesia [2]. There are 397 varieties of black rice around the world, and China accounts for more than 62% of global production [3]. Black rice is often hailed as the “King of Rice”, “Medicinal Rice”, and “Longevity Rice”. It not only has abundant essential nutrients such as amino acids, proteins, vitamins, and minerals, but also contains abundant bioactive components like GABA, anthocyanins, and oryzanol [4]. These ingredients make black rice biologically active in preventing or alleviating the occurrence and development of chronic diseases, such as obesity, alcoholic fatty liver, type 2 diabetes, and gastrointestinal diseases [5,6]. Thus, black rice has attracted increasing popularity worldwide due to its nutritional value and health benefits. However, the dense texture of its husks makes it hard to digest and absorb, and simultaneously difficult for water to penetrate, thereby extending the soaking and cooking time [7]. The high contents of protein and amylose in black rice also result in a rough and non-sticky texture after cooking [8]. Poor palatability and difficulty in steaming and cooking have become the main factors limiting the widespread development and application of black rice in the food industry.

Currently, food processing techniques such as pre-cooking, extrusion puffing, and germination treatment are used to overcome the above problems of whole grains [9]. Germination is a complex process along with numerous biochemical transformations of materials. It has the advantages of low cost, easy operation, and environmental friendliness compared with physical, chemical, and enzymatic treatments. During germination, the respiration rate of seeds increases, accompanied by alterations in seed morphology and cell membrane permeability, significantly activating the activity of endogenous enzymes in seeds [10]. Meanwhile, germination promotes the degradation of food macromolecules (starch, lipids, and proteins). It notably enhances the activity of α-amylase, leading to starch hydrolysis that reduces sugars and other small molecules, along with structural modifications [11]. Furthermore, germination treatment helps to reduce anti-nutritional factors in food, and promotes the release of minerals, thereby improving the digestibility and bioavailability of nutrients [12]. More interestingly, germination can promote the formation and accumulation of plant active components such as GABA and total phenols in grains, leading to enhanced antioxidant activity. It is worth noting that GABA is reported to lower blood pressure, improve brain function, and delay cognitive decline [13]. It has been approved for inclusion in food products in China. Therefore, the germination process can greatly increase the health characteristics of brown rice.

Compared with physical, chemical, and enzymatic treatments, germination attracts more attention because of easy operation, environmental friendliness, high safety, and low cost. During germination, the respiration rate of seeds increases, accompanied by alterations in seed morphology and cell membrane permeability, significantly activating the activity of endogenous enzymes in seeds [10]. Meanwhile, germination promotes the degradation of food macromolecules (starch, lipids, and proteins). It notably enhances the activity of α-amylase, leading to the hydrolysis of starch into reducing sugars and other small molecules, along with structural modifications [11]. Besides, germination treatment helps to reduce anti-nutritional factors in food, promotes the release of minerals, thereby improving the digestibility and bioavailability of nutrients [12]. More interestingly, germination can promote the formation and accumulation of plant active components such as GABA and total phenols in grains, leading to the enhanced antioxidant activity. It is worth noting that GABA is reported to lower blood pressure, improve brain function, and delay cognitive decline [13]. It has been approved for inclusion in food products in China. Therefore, germination process can greatly increase the health characteristics of brown rice.

Additionally, starch is the most significant nutrient component in whole grains, and its water absorption capacity, thermal properties, and gelatinization behavior directly affect the hardness and stickiness of whole grains. Previous studies have found that germination treatment reduced the levels of peak viscosity (PV), final viscosity (FV), and breakdown value (BD), which are negatively correlated with the hardness of rice products [14]. For wheat and brown rice, germination treatment significantly enhanced the oil absorption, foaming, and emulsifying properties of whole flour. Furthermore, it affected the crystallinity, short-range ordered structure, and particle size [15,16]. During the germination, the amounts of rapidly digestible starch (RDS), slowly digestible starch (SDS), and resistant starch (RS) were changed, which are closely related to the digestibility and absorption characteristics of whole grain flour [17]. Therefore, germination is considered as an effective method to improve food quality and enhance its health-promoting potential.

Much research has been focused on germination as a strategy for enhancing the nutritional value and health benefits of whole grain. However, the characteristics of nutritional components, physicochemical properties, and in vitro digestibility of black rice during germination have not been systematically studied. We assumed that the activation of enzymes in black rice would hydrolyze large molecules into smaller molecules during germination, which affect the nutritional components, phytochemical properties, structure, and digestive characteristics, thereby affecting the quality of black rice. Therefore, the nutritional profiles (amylose, reducing sugar, free amino acid, GABA, and minerals), physicochemical properties (water absorption index, water solubility index, thermal properties, pasting properties, relative crystallinity, and long-range order of black rice, functional groups composition), and in vitro digestibility were evaluated.

## 2. Materials and Methods

### 2.1. Material and Chemicals

The black rice used in this experiment was provided by Hunan Hybrid Rice Research Center (Changsha, China). Pepsin (3000 U/g), porcine pancreatic alpha-amylase (290 U/mL), and amyloglucosidase (15 U/mL) were purchased from Yuanye (Shanghai Yuanye Biotechnology Co., Ltd., Shanghai, China). All other chemical reagents applied were of analytical purity.

### 2.2. Preparation of Germinated Black Rice Flour (GBRF)

The germination process was according to the methods described by Cornejo et al. [18] with some modification. Primarily, the black rice was thoroughly cleaned, and the defective grains were removed. They were soaked in 0.1% NaClO at a ratio of 1:2 (*w*/*v*) for 30 min. After being washed several times, they were immersed in distilled water (mass ratio of water to black rice was 5:1) for 12 h at 25 °C. Subsequently, the fully soaked black rice was submitted to the germination incubator under dark, the conditions were set at 28 °C and cyclic spraying for 24 h or 48 h. Finally, the germinated black rice flour (GBRF) was dried at 50 °C for 24 h, and ground into 60 mesh flour for further analysis. The black rice flour (BRF) was served as blank controls.

### 2.3. Analysis of Nutrition Components

#### 2.3.1. Amylose Starch and Reducing Sugar

The amylose starch in GBRF was determined by a colorimetric method according to NY/T 2639-2014 standard methods [19]. In brief, the sample was weighed and mixed with 95% ethanol properly, then 4.5 mL of 0.02% (*w*/*v*) sodium hydroxide solution was added. After gently shaking, the mixed solutions were submitted to boiling for 10 min and diluted with deionized water. Next, 2.5 mL of the above solutions were mixed with 0.5 mL of acetic acid solution and 1 mL of iodine solution. The absorbance was measured at a wavelength of 620 nm after 10 min reaction.

The reducing sugar in GBRF was detected using the 3, 5-dini-trosalicylic acid (DNS) method [20]. Simply, the sample was blended with deionized water for ultrasonic extraction for 30 min. Then, they were centrifugated, and the supernatant was collected and diluted. A total of 3 times the volume of DNS solution was added into the diluted solution. After being boiled for 5 min, they were measured at 540 nm using a UV spectrophotometer (Feile Instrument Co., Ltd., Shanghai, China).

#### 2.3.2. Free Amino Acid and GABA

The content of free amino acids in GBRF was determined using a L-8900 automated amino acid analyzer (Hitachi, Tokyo, Japan). The pretreatment of the samples was referred to the previously reported method with minor revision [21]. Initially, the samples (1 g) were extracted with 50 mL 0.01M HCl for 30 min. Then, they were gently shaken and filtered. A total of 2 mL of sulfosalicylic acid (8%) was added into the supernatant (2 mL). After standing for 15 min, they were subjected to centrifugation at 10,000 rpm for 10 min. Finally, the supernatant was collected and filtered through a 0.45 μm membrane for further analysis.

The detection of GABA in of GBRF was performed on a high-performance liquid chromatograph (HPLC) (Series LC-20/40D 3C, Shimadzu, Japan) equipped with an ultraviolet detector. The procedures used were mainly referred to by Wu et al. [22] with some modification. In brief, the samples were ultrasonically extracted with 80% ethanol solution for 30 min, and centrifuged at 5000 rpm for 5 min. After twice-repeated extraction, the filtrate was combined and diluted to 25 mL with extracting solution. Next, it was treated with derivatization reaction and filtered before HPLC analysis.

#### 2.3.3. Mineral Profiles

The contents of minerals, including sodium (Na), magnesium (Mg), potassium (K), calcium (Ca), iron (Fe), zinc (Zn), and copper (Cu) of GBRF were automatically analyzed using ICP-MS equipment (7850 ICP-MS, Agilent, America). The preparation of samples referred to the method of Wu et al. [23] with proper modification. The samples (0.2 g) were mixed with 10 mL of nitric acid solution (65%) and kept overnight. Then, the combinations were digested on a microwave digestion system. The digested products were diluted to 25 mL with deionized water, and heated at 100 °C for 30 min. The results were expressed in mg/100 g.

### 2.4. Measurement of α-Amylase Activity

The α-amylase activity in GBRF was determined according to Luo et al. [15] with minor revision. The samples (0.50 g) were dissolved in 10 mL deionized water, and incubated for 20 min. After being centrifuged at 8000 rpm for 15 min, the supernatant was obtained and filtered. The filtrate was placed in a 70 °C water bath for 15 min to inactivate the β-amylase and then kept balanced at 40 °C for 10 min. Followingly, the filtrate (1 mL) was mixed with 1 mL of 1% (*w*/*v*) soluble starch solution, and reacted at 40 °C for 5 min. A 0.4 M sodium hydroxide solution was used to terminate the reaction. Finally, 2 mL of DNS reagent was added, and was submitted to boiling for 10 min. The absorbance was measured at 540 nm after cooling and dilution. The α-amylase activity (U/g) was expressed as mg glucose/g dry weight per minute at 40 °C.

### 2.5. Water Absorption Index (WAI) and Water Solubility Index (WSI)

The WAI and WSI of GBRF were determined according to the method published by Lee et al. [24] with some adjustment. The samples (0.2 g) were evenly dispersed in 10 mL deionized water by being fully shocked. After being kept at 30 °C for 30 min, the mixed solutions were gently stirred every 5 min. Then, they were centrifuged at 3000 r/min for 20 min to obtain the precipitation. Furthermore, the supernatant was dried at 105 °C to a constant weight. WAI and WSI were calculated as below:

(1) $$WAI(\%)=\frac{m_{1}}{m_{0}}\times 100$$

(2) $$WSI(\%)=\frac{m_{2}}{m_{0}}\times 100$$

where *m*_0_ represents the mass of the samples; *m*_1_ represents the mass of the precipitation; and *m*_2_ was the mass of the supernatant after being dried to constant weight.

### 2.6. Differential Scanning Calorimetry (DSC) Analysis

The gelatinization properties of GBRF were analyzed using a DSC (Q20, TA Instruments, New Castle, DE, USA) under nitrogen purge atmosphere [20]. The samples (5 mg) were accurately weighed and blended with 10 µL deionized water in a stainless steel plate. After being hermetically sealed, they were equilibrated at 4 °C for 24 h, and heated from 30 °C to 95 °C at a rate of 10 °C/min. The transition temperature, including onset temperature (To), peak temperature (Tp), end temperature (Tc), and enthalpy (ΔH), were measured according to the DSC heating curves.

### 2.7. Rapid Visco Analyzer (RVA) Analysis

The pasting properties of GBRF were analyzed by a Rapid Visco Analyzer (RVA 4500, Perten Instruments, Stockholm, Sweden), as referred to previously in the method of Luo et al. [15]. The samples (3.000 ± 0.001 g) were placed in an aluminum RVA box, and the proper amount of deionized water was added to reach a total weight of 28.00 g. The temperature program was set as follows: the mixture was equilibrated at 50 °C for 1 min and heated from 50 °C to 95 °C at 6 °C/min, then kept at 95 °C for 5 min and cooled to 50 °C at 6 °C/min, finally being maintained and held at 50 °C for 2 min. The pasting parameters, including peak viscosity (PV), trough viscosity (TV), breakdown value (BV), final viscosity (FV), and setback value (SV), were measured.

### 2.8. In Vitro Digestibility Test

The digestive capacity of GBRF was conducted in a simulated environment according to the procedures outlined by Nie et al. [25] with minor modification. In the first stage, the samples (50 mg) and deionized water (2 mL) were added to the centrifuge tubes with 2 glass beads. The centrifuge tubes were kept boiling in water for 10 min, and cooled to 37 °C. Then, the samples were incubated at 150 rpm/min at 37 °C for 30 min after HCl buffer (2 mL, pH 2) and pepsin (1 mg, 3000 U/g) was added. In the second stage, the primary enzymatic hydrolysate was blended with sodium acetate buffer (4 mL, pH 5.2) and mixed enzyme solution (1 mL), which consisted of porcine pancreatic α-amylase (290 U/mL) and amyloglucosidase (15 U/mL). Furthermore, the sample tubes were incubated in the same conditions as before. During the incubation, the enzymolysis solutions (1 mL) were collected at intervals of 0, 20, and 120 min and 95% ethanol (4 mL) was added to terminate the enzymatic reaction. After centrifugation, the supernatant was gathered and the DNS solution was used to test the glucose content. The values of RDS, SDS, and RS were calculated through the following formulas:

(3) $$RDS(\%)=\frac{\left(G_{20}-G_{0}\right)}{TS}\times 0.9\times 100$$

(4) $$SDS(\%)=\frac{\left(G_{120}-G_{20}\right)}{TS}\times 0.9\times 100$$

(5) RS(%)=1−RDS−SDS

where *G*_0_, *G*_20_, and *G*_120_ represent the glucose content digested at 0, 20, and 120 min, respectively, TS represents the total starch in the original sample, and 0.9 was the converting factor of glucose to starch.

### 2.9. Analysis Methods of Structural Properties

#### 2.9.1. Scanning Electron Microscopy (SEM)

The microstructure of GBRF was observed by scanning electron microscopy (TM3000, Hitachi, Japan). The final moisture content of all the samples was controlled to less than 8%. The samples were coated with gold–palladium, and examined at an acceleration voltage of 10 kV.

#### 2.9.2. Fourier Transform Infrared Spectroscopy (FTIR)

FTIR characterization (Cary 630 Agilent Technologies, Santa Clara, CA, USA) was performed to acquire the spectrum of GBRF by the protocol of Rahaman et al. [26] The lyophilized GBRF was fully mixed with dry KBr powder at a ratio of 1:100, and the mixture was pressed into thin slice, and detected in the 4000–400 cm^−1^ range.

#### 2.9.3. X-Ray Diffraction (XRD)

X-ray diffraction analysis of GBRF was determined using a Shimadzu XRD 7000 diffractometer (Shimadzu Corporation, Kyoto, Japan). The measurement was operated at 40 kV and 40 mA. The scanning of the samples was captured in the range of 5–50° (2θ) at the speed of 2 °/min. The relative crystallinity (RC) was analyzed by the ratio of area under crystalline peaks to the total spectral area for each sample according to Zhang el al. [27].

### 2.10. Statistical Analysis

All the data were measured in triplicate, and are shown as means ± standard deviation (SD). Origin software 2024 and graphpad prism 8. were used to draw the graphs. A one-way ANOVA test and Duncan multiple comparison tests (*p* < 0.05) were used for statistical analysis.

## 3. Results and Discussion

### 3.1. The Effect of Germination on the Apparent and Microscopic Characteristics of BRF

**Figure 1:**
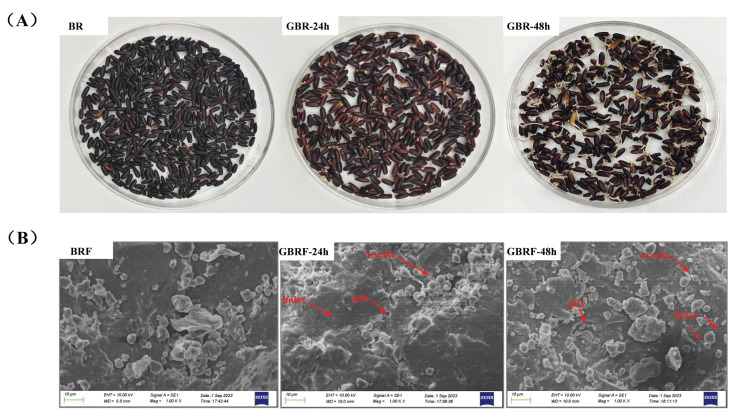
Macroscopic and microscopic structure of black rice (BR) and black rice flour (BRF) during different germination periods. (**A**) appearances of BR, GBR-24 h, and GBR-48 h; (**B**) scanning electron micrographs of BRF, GBRF-24 h, and GBRF-48 h (×1000).

In present, macroscopic and microscopic characteristics of germinated black rice flour were observed to explore the effect of germination on the structure of black rice (Figure 1). As shown in Figure 1A, we found that germination rate and sprout length increased significantly with the prolongation of germination time. When germinated for 48 h, the sprout was long enough and not suitable for further germination. Moderate germination promotes the production of bioactive components in grains and improves the nutritional value of grains, while excessive germination leads to a loss of nutrients and the production of unpleasant odor, thus affecting its consumption [28,29]. Lan et al. [30] studied the germination of *Chenopodium quinoa* Willd. for 0–72 h, and the results confirmed that 12–48 h germination significantly increased the total free amino acids and reduced the undesirable volatile components. Absolutely, the germination process is affected by the types of raw materials and the processing technology. Figure 1B exhibits the micro-morphological structure of germinated black rice flour using scanning electron microscopy (SEM) at 1000× magnification. It was found that the surface of BRF was relatively dense and smooth, with less cracks, pits, or pores. When germinated for 24 h, cracks appeared on the surface of BRF, and a few small pits and pores can be seen. With the germination time extended to 48 h, more cracks, pits, and pores were observed, compared with black rice and germinated black rice for 24 h. These pores and cracks offered a channel for the endogenous amylase to enter the starch granules, thus promoting the hydrolysis of the starch granules by fully contacting with hydrolase [14]. Similar phenomena also appeared on waxy brown rice [14], oat, sorghum, and millet [31] after germination. Germination can make the dense structure of black rice looser and softer, and improve its overall edible property.

### 3.2. The Effect of Germination on Nutritional Profiles of BRF

#### 3.2.1. Changes of Apparent Amylose and Reducing Sugar During Germination

**Figure 2:**
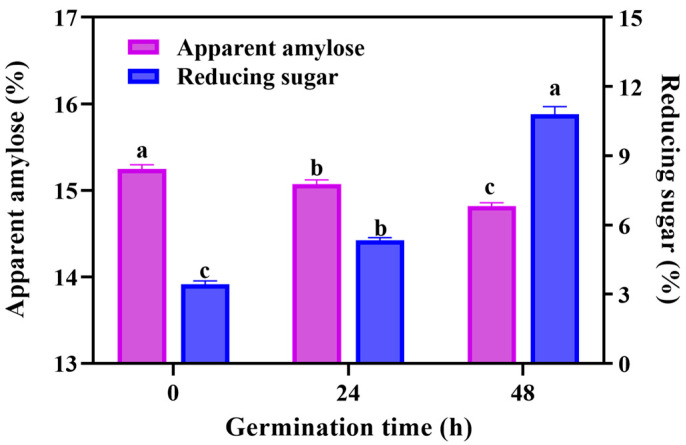
The effect of germination on the content of apparent amylose and reducing sugar during germination. Data are presented as mean ± SD (*n* = 3). Values with different letters indicate significant differences *p* ≤ 0.05.

Germination is a cell life activity of seeds, accompanied with the conversion of nutrients and the flow of energy. Therefore, we firstly analyzed the changes of apparent amylose and reducing sugar in GBRF. As can be seen in Figure 2, the amylose content of GBRF declined during the germination, ranging from 15.25 ± 0.05% to 14.82 ± 0.04%. A nutritional study of *Yoom Noon* rice displayed that the white rice had approximately 24% amylose, followed by brown rice with 22% and germinated brown rice with 20%, respectively (*p* < 0.05) [32]. This result indicated that germination treatment promoted amylose metabolism. A similar trend was also reported by Wang et al. [33] in the study of brown rice germination. However, a study [14] found that germination did not obviously alter the apparent amylose content of starch of waxy rice, suggesting that the change of apparent amylose content may be affected by raw materials during germination.

In contrary, the reducing sugar content increased remarkably with the time of germination. The reducing sugar content of BRF was 3.42%. After 24 h and 48 h germination, it increased to 1.56 times and 3.16 times, respectively. This trend kept in line with previous studies. Kim et al. [21] reported that the reducing sugar content in brown rice significantly increased from 3224.06 mg/100 g to 5176.14 mg/100 g after 2 days of germination, and then slightly decreased after 3 days of fermentation. The decline can be explained, in that the generated reducing sugars enter other metabolic pathways as germination continues, and then the consumption exceeds the production [34]. Reducing sugar is the primary metabolite of hydrolysis of residue starch, as it not only provides the energy for the black rice to germinate, but also contributes to improving the taste of black rice.

#### 3.2.2. Changes of Free Amino Acid and GABA During Germination

**Figure 3:**
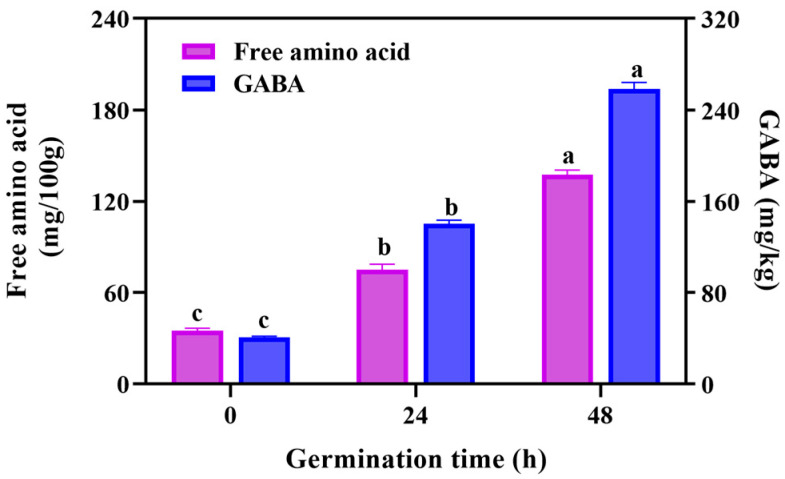
The effect of germination on the content of free amino acid and γ-aminobutyric acid (GABA) during germination. Data are presented as mean ± SD (*n* = 3). Values with different letters indicate significant differences *p* ≤ 0.05.

Free amino acid is an important nutritive index of grain-based foods. Through analyzing the amino acids (Figure 3) of black rice during germination, we discovered that the free amino acid content of black rice showed a significant upward trend during 0–48 h germination, increasing from 34.97 mg/100 g to 137.40 mg/100 g, respectively. Similar results were observed in the brown rice germination of Kim et al. [21] It can be explained that germination activates the residual enzymes responsible for the hydrolysis of proteins to produce peptides or free amino acids [21]. Liu et al. [10] declared that the protease activity markedly enhanced during maize germination. The hydrolysis of proteins make it easier for digestion, and abundant amino acids of GBRF fortified the nutritional value of black rice.

Gamma-aminobutyric acid (GABA) is a functional non-protein amino acid in brown rice, which has biological activities such as improving cognitive functions and lowering blood pressure [35,36]. During germination, the residual glutamate of rice may be converted to GABA by glutamate decarboxylase [37]. In this study, the GABA content showed an increasing trend with the extension of germination. When germinated for 48 h, the GABA content was 258.35 mg/kg, which was 6.34 times and 1.84 times higher than those of 0 h and 24 h, respectively. It was proved that germination was an efficient way to obtain GABA of brown rice. In addition, germinated treatment also significantly promoted GABA accumulation in wheat [16], quinoa [30], sunflower seeds [38], and buckwheat [39]. These results indicate that germination is an efficient and eco-friendly strategy to enrich the phytochemicals, for example, GABA.

#### 3.2.3. Changes of Minerals During Germination

Minerals are required nutrients for seed germination, and they are mainly distributed in the outer cortex of grains. To observe the effect of germination on the mineral content of black rice, the contents of seven kinds of minerals were examined, including Na, Mg, K, Ca, Fe, Cu, and Zn. As shown in Table 1, the contents of Na, Ca, and Cu were remarkably increasing with germination time, and they varied from 12.33 to 32.93 mg/kg, 45.90 to 91.47 mg/kg, and 0.28 to 0.41 mg/kg, respectively. Rice bran is an important resource of minerals, accounting for about 61% [40]. Generally, minerals are observed in the form of complexes with phytic acid or fiber in the seed. Germination can significantly enhance the bioavailability of some minerals through the hydrolysis of its bound state with phytic acid [41]. Therein, calcium is known as an important regulator of amylases and proteases, involving in the enzymatic catalytic reactions [41]. Sodium is able to regulate enzyme reaction, maintain osmotic pressure, and promote muscle contraction [42]. A higher content of Ca and Na were present in germinated white quinoa, while the content of Na decreased in germinated red quinoa [43]. Moreover, the contents of Mg, Fe, and Zn increased first and then decreased after germination. Their contents reached the highest when germinated at 24 h, namely 1435.63 mg/kg, 13.03 mg/kg, and 17.37 mg/kg, respectively. In the germination of sunflower seed, the contents of Ca and Mg reached the highest at 48 h and 24 h, separately [38]. On the contrary, K content decreased from 4044.93 mg/kg to 3148.07 mg/kg during the germination. It was also investigated by Jan et al. [42] that K content of *Chenopodium album* showed a marked decline from 10,113.31 mg/kg to 3021.7 mg/kg after 48 h germination. The reduction in parts of minerals may be related to the leakage caused by pre-germination soaking, or utilization in the development of seeds. In short, germination stimulated the variation of minerals and improved its bioavailability.

### 3.3. The Effect of Germination on the Physicochemical Properties of BRF

Germination is a cell life activity of seeds, accompanied with the conversion of nutrients and the flow of energy. It mainly induced the activation of endogenous hydrolase, such as α-amylase, β-amylase, glucosidase, etc., which promoted the hydrolysis of the starch to some extent. Then, we analyzed the effects of the germination process on the physicochemical properties of GBRF, including α-amylase activity, WAI, and WSI. As can be seen in Table 2, the black rice had the lowest α-amylase activity of 1.02 U/g. As germination continues, the α-amylase activity increased to 2.05 U/g and 4.46 U/g for 24 h and 48 h, respectively. In the germination process of wheat flour, its total amylolytic activity was observed to have a pronounced increase, especially α-amylase, from 12.62 U/g of non-germination to 17.52 U/g of 72 h germination [16]. The α-amylase activity of germinated brown rice reached the highest of 6.6 U/g at 48 h, and the content of reducing sugar was markedly increased [15]. Equally, germination induced an increase of 3.9 times of amylase activity and 2.4 times of reducing sugar of maize at 48 h, along with a decrease of 21.48% of starch [10]. Of course, β-amylase and protease activity were also observed to be increased during the germination, which promoted the degradation of starch and protein [10,14]. The above results indicated that enzymes, especially α-amylase, play a key role in the process of grain germination.

WAI and WSI reflected the interaction of polymer chains composed of amorphous and crystalline particles. A higher WAI value suggests a stronger capacity of starch to absorb water, which is mainly affected by the quantity of hydrophilic groups in the flour [44]. The WAI of GBRF decreased with time, from 2.58 g/g (0 h) to 2.29 g/g (48 h), which keeps the same trend with that of wheat flour during germination [16]. During germination, dextrin and fermentable sugars are produced by the hydrolysis of carbohydrates in black rice, which promote the formation of cross-links with the amorphous regions of starch chains [45]. As a result, the WAC (water absorption capacity) decreases. Inversely, WSI continually increased with time, from 1.61% (0 h) to 3.08% (48 h). These changes may be attributed to the breakdown of starch by α-amylase, leading to the generation of small molecules dissolving in aqueous solutions.

### 3.4. The Effect of Germination on the Thermal Properties of BRF

The thermal properties of BRF during different germination periods were evaluated by DSC, including To, Tp, Tc, and ∆H. The parameters are related to the dissociation of ordered molecular structure and the amount of double helical structure [14]. As shown in Table 3, the To value of the samples varied between 78.67 and 80.03, the Tp value varied between 83.49 and 83.61, and the Tc value varied between 87.55 and 87.80. However, there were no significant differences in these parameters in each group (*p* > 0.05). Germination notably increased the ∆H value of samples, in which GBRF-24 h has the highest ∆H value of 12.27 ± 0.09 J/g, higher than that of GBRF-48 h (12.05 ± 0.03 J/g) and GBRF-0 h (10.57 ± 0.09 J/g). Similarly, Luo et al. [15] found that the ∆H value of germinated brown rice starch was significantly higher than that of ungerminated brown rice starch. In the research of germination influence on the gelatinization properties of brown rice, oat, sorghum, and millet, Li et al. [31] found that the To and Tc values of brown rice and millet increased with the prolonging of germination time, whereas the To value of oat and the Tc value of sorghum showed no significant variation. The values of ∆H of brown rice exhibited a significant decrease during germination, while the ∆H value of sorghum and millet showed a significant increase. The differentiated results might be ascribed to the content of amylose, microstructure, particle size distribution, and the amylose/amylopectin ratio of grains [46].

### 3.5. The Effect of Germination on the Pasting Properties of BRF

Pasting properties are the important indicators to measure the qualities of starch-based products. It is usually evaluated with PV, TV, BV, FV, and SV. As illustrated in Table 4, the values of peak viscosity, trough viscosity, breakdown viscosity, final viscosity, and setback viscosity remarkably decreased during the germination, and the downtrend was more obvious with time. These parameters ranged from 2037.67 to 2932, 942.67 to 1810.33, 1095.00 to 1121.67, 1519.67 to 2601.67, and 577.00 to 791.33, respectively. Our results found that the PV of GBRF-48 h reduced to 30.50% compared to the BRF. This may be due to the hindering of the swelling ability of GBRF by germination, thereby limiting the interaction between starch and water, and finally leading to the reduction in PV. Furthermore, the decrease in PV by germination would be due to the digestion of starch by activated α-amylase. Consistently, the TV, FV, and SV of GBRF decreased by 47.93%, 41.59%, and 27.08% at 48 h germination, respectively, which illustrated that the starches in GBRF were less resistant to high temperature and shearing. The BV of GBRF reached the highest at 24 h germination, and then reduced at 48 h. Similarly, Ukpong et al. [47] found that different varieties of brown rice exhibited different pasting properties. The PV, TV, FV, and SV of the all the samples presented a downward trend within 12–36 h of germination. Both wild-type brown rice and gene knockout brown rice showed a significant decrease in PV, TV, BV, FV, and SV after 48 h of germination [34]. Generally speaking, the changes in the pasting property of germinated brown rice are mainly attributed to the hydrolysis of starch to small molecules by the activated endogenous amylase, leading to the significant viscosity reduction.

### 3.6. The Effect of Germination on In Vitro Digestibility of BRF

**Figure 4:**
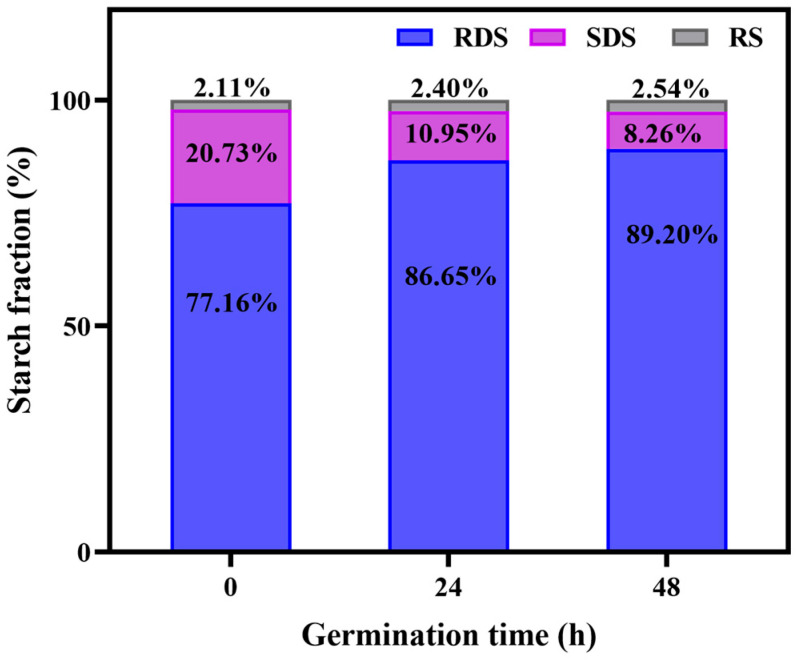
Starch in vitro digestion of black rice flour (BRF) during different germination periods.

Nutrients in food, such as starch and protein, are the biological macromolecules. They usually need to be digested into small molecules through the digestive tract before they can be absorbed and utilized by the human body. The digestibility of starch in grains directly affect human health. Based on the rate and degree of digestion, starch can be classified into three types, namely RDS, SDS, and RS [26]. To explore the effect of germination on the in vitro digestion of GBRF, the percentages of RDS, SDS, and RS were examined and shown in Figure 4. For BRF, RDS accounts for the largest proportion of 77.16%, followed by SDS (20.73%) and RS (2.11%). The RDS content remarkably increased at 48 h germination, rising by 12.04%. RDS can be rapidly hydrolyzed in the mouth and small intestine to provide the energy needed by the body. However, the intake of high RDS in grains could cause a rapid rise in blood sugar and the insulin response disorders for special populations such as diabetics, obesity, etc. In contrary, the content of SDS shows a significant downward trend during 48 h germination, decreasing from 20.73% to 8.26%. The digestion of SDS mainly occurs in the small intestine, where its digestion and absorption are relatively slow, and the glucose is released slowly. The intake of SDS in grains can prevent a rapid increase in blood sugar and help to prevent various chronic diseases. RS is hard to digest and absorb, simultaneously having a slow release. It cannot be degraded and absorbed in the small intestine, but it could be utilized by the gut microbiota in the colon. RS plays an important role in regulating blood sugar balance and maintaining gut health. Our research confirmed that the RS content showed a certain upward trend with the prolonging of germination. The above results indicate that germination alters the composition of starch, and the germination time may directly affect the digestibility of starch.

### 3.7. XRD

**Figure 5:**
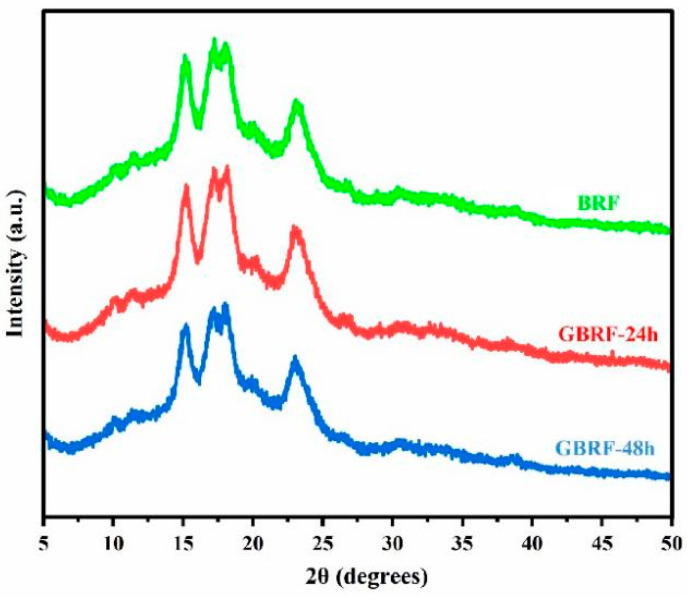
X-ray diffraction patterns of black rice flour (BRF) during different germination periods.

Starch molecules showed a semi-crystalline state, generally composed of the crystalline region of amylopectin and the amorphous region of amylose. At present, X-ray diffraction analysis is usually used to explore crystalline conformation. Natural starch can be classified into three types of A, B, and C according to the XRD analysis [13]. The variation of the crystal structure of GBRF was tested and shown in Figure 5. Notably, all samples presented the type-A crystal pattern of grain starch, with characteristic diffraction peaks at 15°, 17°, 18°, and 23°. The position of the diffraction peak of black rice starch did not alter significantly before and after germination, indicating that the crystal type of starch did not change during germination. This may be related to the compactly arranged double helix of type-A structure in starch [46]. To further clarify the variation of the crystal structure, the analysis of relative crystallinity (RC) was performed. Interestingly, we found that germination induced a decreasing trend of RC. The RC of BRF was 32.30% ± 1.15%, significantly higher than RC of GBRF-24 h (31.47% ± 0.87%), and GBRF-48 h (29.86% ± 0.90%), respectively. Similar results were reported in glutinous rice [14] and brown rice [48] with germination time. It might be the fact that the connection and winding of the double helix structure of starch was disrupted during the germination, resulting in the formation of irregular crystalline structure by the hydrolysis of starch particles.

### 3.8. FTIR

**Figure 6:**
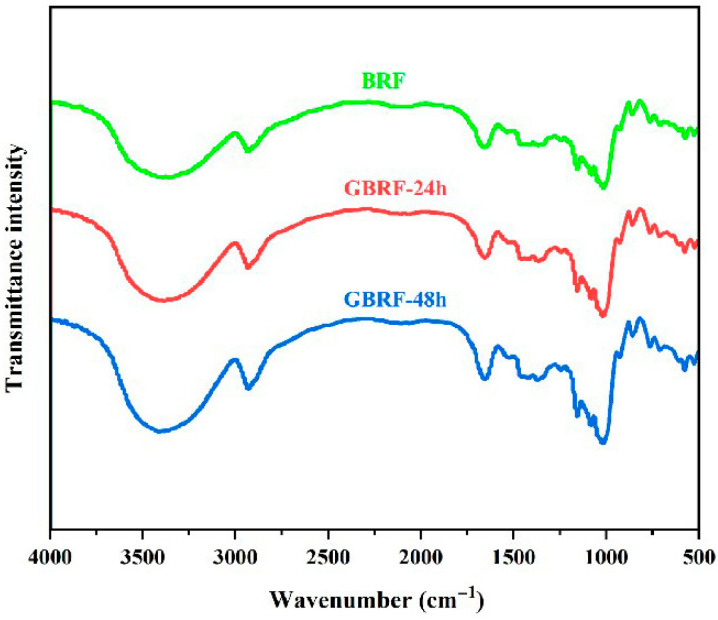
FTIR spectra of black rice flour (BRF) during different germination periods.

FTIR was an efficient method to analyze the composition of functional groups in ingredients. As shown in Figure 6, the starches of black rice flour and GBRF exhibited characteristic absorption peaks of grain starch. At the band of 3200–3500 cm^−1^, there was a strong absorption peak relating to the stretching vibration of -OH. The band of 2930 cm^−1^ responded to the stretching vibration of -CH, and the absorption peak at 1657 cm^−1^ was caused by the presence of amide and water. Meanwhile, hydrogen bonds in the hydroxyl group resulted in the absorption peak at 1000 cm^−1^. It is worth mentioning that the sensitive band of starch molecules is distributed in 1300 cm^−1^–800 cm^−1^, and the stretching vibration of C-O and C-C bonds mainly occurs in this region. As a whole, the FTIR spectra of the starches of BRF and GBRF presented similar stretching vibration under different germination times, only existing with some differences in absorption intensity, which indicated that no new functional groups were generated during the germination process.

## 4. Conclusions

The influence of germination on nutritional components, physicochemical properties, structure variations, and in vitro digestibility of black rice flour were investigated in this study. Compared with BRF, the basic nutritional components of GBRF were fortified during germination, including reducing sugar, free amino acids, and minerals. It is worth noting that the GABA content was significantly increased by 6.3 folds when compared with BRF. Water absorption of BRF was decreased and water solubility was increased during the germination period. Moreover, GBRF was observed as a looser structure, a lower relative crystallinity and lower viscosity than BRF. These characteristics make it more suitable for food processing by improving the dispersion and weakening the abrasive physical forces to the equipment. Furthermore, we found that germination improved the digestibility of black rice by increasing the content of RDS and reducing the content of SDS. This could be applied in cookies, bread, and beer production with health benefits [49]. However, there are still many issues that need to be further explored, such as the molecular weight distribution and chain length changes of black rice starch during the germination, the interaction relationships between starch hydrolysis and the active components, etc..

## Figures and Tables

**Table 1:** Mineral changes of BRF during different germination periods.

Sample	Na (mg/kg)	Mg (mg/kg)	K (mg/kg)	Ca (mg/kg)	Fe (mg/kg)	Cu (mg/kg)	Zn (mg/kg)
BRF	12.33 ± 0.71 ^c^	1231.07 ± 43.72 ^b^	4044.93 ± 93.16 ^a^	45.90 ± 4.25 ^c^	11.83 ± 0.65 ^a^	0.28 ± 0.03 ^b^	13.77 ± 0.85 ^b^
GBRF-24 h	29.13 ± 1.65 ^b^	1435.63 ± 69.75 ^a^	3727.83 ± 77.64 ^b^	81.30 ± 6.83 ^b^	13.03 ± 0.76 ^a^	0.38 ± 0.01 ^a^	17.37 ± 1.00 ^a^
GBRF-48 h	32.93 ± 0.65 ^a^	1272.07 ± 73.39 ^b^	3148.07 ± 63.57 ^c^	91.47 ± 2.51 ^a^	11.90 ± 0.89 ^a^	0.41 ± 0.01 ^a^	14.60 ± 0.56 ^b^

Note: All the results are presented as mean ± SD (*n* = 3). Superscript letters (a–c) in the same row represent statistically significant differences at *p* ≤ 0.05 and the same superscript letter indicates no significant difference (*p* > 0.05).

**Table 2:** Physicochemical property changes of BRF during different germination periods.

Sample	α-Amylase Activities (U/g)	WAI (g/g)	WSI (%)
BRF	1.02 ± 0.01 ^c^	2.58 ± 0.02 ^a^	1.61 ± 0.03 ^c^
GBRF-24 h	2.05 ± 0.06 ^b^	2.39 ± 0.03 ^b^	2.25 ± 0.06 ^b^
GBRF-48 h	4.46 ± 0.02 ^a^	2.29 ± 0.03 ^c^	3.08 ± 0.06 ^a^

Note: The data are expressed as mean ± SD (*n* = 3). Different lowercase letters in the same column represent significant difference at *p* < 0.05.

**Table 3:** Thermal characteristics of BRF during different germination periods.

Sample	To (°C)	Tp (°C)	Tc (°C)	∆H(J/g)
BRF	80.03 ± 0.27 ^a^	83.60 ± 0.13 ^a^	87.80 ± 0.17 ^a^	10.57 ± 0.09 ^c^
GBRF-24 h	78.67 ± 1.12 ^a^	83.61 ± 0.10 ^a^	87.68 ± 0.11 ^a^	12.27 ± 0.09 ^a^
GBRF-48 h	80.02 ± 0.18 ^a^	83.49 ± 0.22 ^a^	87.55 ± 0.08 ^a^	12.05 ± 0.03 ^b^

Note: All the results are expressed as mean ± SD (*n* = 3). Superscript letters (a–c) in the same row represent statistically significant differences at *p* ≤ 0.05, and the same superscript letter indicates no significant difference (*p* > 0.05). To: onset temperature; Tp: peak temperature; Tc: conclusion temperature; ∆H: enthalpy change.

**Table 4:** Pasting properties of BRF during different germination periods.

Sample	Peak Viscosity (cP)	Trough Viscosity (cP)	Breakdown Viscosity (cP)	Final Viscosity (cP)	Setback Viscosity (cP)	Peak Time (min)	Pasting Temperature (°C)
BRF	2932.00 ± 30.15 ^a^	1810.33 ± 32.56 ^a^	1121.67 ± 6.51 ^b^	2601.67 ± 46.26 ^a^	791.33 ± 14.47 ^a^	5.80 ± 0.01 ^a^	85.17 ± 0.06 ^a^
GBRF-24 h	2739.00 ± 44.51 ^b^	1559.33 ± 38.76 ^b^	1179.67 ± 13.05 ^a^	2320.00 ± 46.51 ^b^	760.67 ± 14.29 ^a^	5.71 ± 0.01 ^b^	85.30 ± 0.10 ^a^
GBRF-48 h	2037.67 ± 39.02 ^c^	942.67 ± 25.48 ^c^	1095.00 ± 20.07 ^b^	1519.67 ± 54.72 ^c^	577.00 ± 32.08 ^b^	5.28 ± 0.02 ^c^	84.31 ± 0.04 ^b^

Note: All the results are expressed as mean ± SD (*n* = 3). Superscript letters (a–c) in the same row represent statistically significant differences at *p* ≤ 0.05, and the same superscript letter indicates no significant difference (*p* >0.05).

## Data Availability

The original contributions presented in this study are included in the article. Further inquiries can be directed to the corresponding author.
